# Editorial: Impact of soil health on nutritional quality of crops and human health

**DOI:** 10.3389/fnut.2026.1790443

**Published:** 2026-02-06

**Authors:** Tarun Belwal, Durgesh K. Jaiswal, Sapna Langyan

**Affiliations:** 1 7 Continents Natural Products, South Brunswick, NJ, United States; 2 Department of Biosciences, Graphic Era (Deemed to be University), Dehradun, India; 3 Division of Germplasm Evaluation, Indian Council of Agricultural Research (ICAR)-National Bureau of Plant Genetic Resources, New Delhi, India

**Keywords:** crop yields, food safety, nutritional outcomes, society affiliation RT, soil health, soil management, soil microbes

Soil health underpins the sustainability of global food systems and plays a decisive role in determining crop productivity, food quality, and human health. Healthy soils function as dynamic living systems in which biological, chemical, and physical processes interact to regulate nutrient cycling, plant growth, and resilience to environmental stress. When soils are degraded through erosion, nutrient depletion, contamination, or loss of biological diversity, crops often exhibit reduced nutritional quality, contributing to micronutrient deficiencies and increased risks of malnutrition. Conversely, soils rich in organic matter and microbial diversity promote healthier crops with enhanced nutrient density, reinforcing the close link between soil condition and human wellbeing.

Beyond nutrition, soil health is closely tied to food safety, ecosystem stability, and agricultural resilience. Beneficial soil microorganisms suppress plant pathogens, reduce disease incidence, and improve nutrient use efficiency, thereby supporting safer and more sustainable food production. In the context of climate change, biodiversity loss, and intensifying land use, improving soil health has emerged as a nature-based solution to enhance food security while mitigating environmental and climate-related risks.

This Research Topic, *Impact of Soil Health on Nutritional Quality of Crops and Human Health*, was developed to explore the interconnected relationships between soil properties, crop nutritional quality, and human health outcomes. The Research Topic brings together original research and review articles that examine how soil management practices, such as balanced nutrient inputs, organic amendments, microbial interventions, and climate-adaptive strategies, can enhance soil functionality and crop nutritional profiles. The studies brought together in this Research Topic collectively tell this deeper story, one in which soil health emerges as a central protagonist in the global quest for food quality, nutritional security, and human wellbeing.

The story begins in the highly weathered soils of Sub-Saharan Africa, where sulfur, often overlooked in fertilizer regimes, quietly governs both soil fertility and the nutritional value of staple crops. Moshi et al. synthesize decades of evidence to show that sulfur deficiency is not merely a soil constraint but a nutritional bottleneck, linking soil degradation directly to hidden hunger. Their work reminds us that restoring balance to soil nutrients can simultaneously rehabilitate soils and improve the quality of diets in regions where resources are limited but needs are immense.

From nutrient-depleted soils, the narrative moves to climates in flux. In tropical Colombia, Beltran-Medina et al. reveal how soil properties interact with climate variability, particularly ENSO-driven fluctuations, to regulate crop evapotranspiration and productivity in basil. Here, soil is not a passive medium but an active moderator of climate stress, influencing how plants access and use water. This study underscores a critical lesson for a warming world: sustainable crop quality will depend on adaptive soil and water management strategies that acknowledge the inseparable link between climate and edaphic conditions.

As productivity and climate resilience are considered, the story turns to food safety, an equally vital but often underappreciated dimension of soil health. Koley et al. explore how microbially mediated silicon-based agro-wastes can reduce arsenic bioaccumulation in crops. Their work highlights the power of soil microbial processes and targeted amendments to interrupt the transfer of toxic elements from soil to plant to plate. In doing so, it reframes soil management as a frontline defense for public health, not merely an agronomic intervention.

Measuring and safeguarding crop quality, however, requires tools that can keep pace with the complexity of soil–plant interactions. Sharma et al. introduce near-infrared spectroscopy as a high-throughput, non-destructive approach for assessing key nutritional traits in oilseed Brassica species. Their study bridges the gap between soil management and food composition, enabling rapid evaluation of nutritional outcomes that were once costly and time-consuming to measure. This technological advance strengthens our ability to translate soil health into tangible improvements in food quality.

The narrative deepens further into the living soil, where biological diversity shapes plant health in profound ways. Amrutha Lakshmi et al. demonstrate how Trichoderma species suppress Ganoderma-induced basal stem rot in oil palm, offering mechanistic insights into microbial-based disease control. Their findings reinforce the idea that resilient soils, rich in beneficial microbes, can protect crops naturally, reducing dependence on chemical inputs while sustaining long-term productivity.

The story comes full circle with Chandel et al., who disentangle the role of native soil microbes in enhancing both soil health parameters and the accumulation of nutritionally valuable phytochemicals in Aloe vera. This work vividly illustrates the soil–microbe–plant nexus, showing how invisible biological communities beneath our feet shape the nutritional and functional quality of crops consumed by humans.

Together, the articles in this Research Topic demonstrate that soil health is not merely an agronomic concern but a key determinant of food quality, food safety, and human nutrition. By integrating perspectives from soil science, agronomy, microbiology, plant nutrition, and public health, this Research Topic underscores the need for holistic, systems-based approaches to agricultural management. We hope this Research Topic will stimulate interdisciplinary research, inform sustainable land-use policies, and encourage the adoption of soil management practices that enhance crop nutritional quality and support human health.

